# Resveratrol as a Tumor-Suppressive Nutraceutical Modulating Tumor Microenvironment and Malignant Behaviors of Cancer

**DOI:** 10.3390/ijms20040925

**Published:** 2019-02-20

**Authors:** Youngjin Han, HyunA Jo, Jae Hyun Cho, Danny N. Dhanasekaran, Yong Sang Song

**Affiliations:** 1 Biomodulation, Department of Agricultural Biotechnology, Seoul National University, Seoul 08826, Korea; youngjin.han@snu.ac.kr (Y.H.); whgusdk25@snu.ac.kr (H.J.); 2 Cancer Research Institute, College of Medicine, Seoul National University, Seoul 03080, Korea; 3 Department of Obstetrics and Gynecology, College of Medicine, Seoul National University, Seoul 03080, Korea; chojhn@gmail.com; 4 Stephenson Cancer Center, The University of Oklahoma Health Sciences Center, Oklahoma city, OK 73012, USA; danny-dhanasekaran@ouhsc.edu

**Keywords:** resveratrol, tumor microenvironment, cancer, chemoresistance, metastasis

## Abstract

Tumor-suppressive effects of resveratrol have been shown in various types of cancer. However, regulation of tumor microenvironment by resveratrol is still unclear. Recent findings suggest resveratrol can potentiate its tumor-suppressive effect through modulation of the signaling pathways of cellular components (fibroblasts, macrophages and T cells). Also, studies have shown that resveratrol can suppress malignant phenotypes of cancer cells acquired in response to stresses of the tumor microenvironment, such as hypoxia, oxidative stress and inflammation. We discuss the effects of resveratrol on cancer cells in stress environment of tumors as well as interactions between cancer cells and non-cancer cells in this review.

## 1. Introduction

Plant-derived compounds are perceived to be less harmful and expected to show minimal toxicity when used as a therapeutic agent. Until now, a number of studies have reported the tumor-suppressive properties of resveratrol which could be isolated from peanuts, grapes and berries [1,2,3,4,5]. However, little is known about the effect of resveratrol on the regulation of various factors existing in the tumor microenvironment. Although many pre-clinical studies have shown the tumor-suppressive effects of various phytochemicals on cancer cells, most of them failed in clinical trials [6]. The clinical trials of resveratrol have focused primarily on colon and rectal cancer and have been performed on phase 1 or 2 of clinical studies, confirming the pharmacological effects and efficacy of resveratrol. Although phase 3 study has not been conducted yet, the combination of conventional chemotherapeutic agents or novel targeted therapy agents with resveratrol has suggested possibilities of resveratrol for the treatment of refractory cancer (Table 1).

In order to obtain a full grasp on the efficacy of a plant-derived compound as a tumor-suppressive agent, the effect of the compound should be tested and recognized not only in cancer cells but also in non-cancer cells that are important constituents of the tumor microenvironment. In addition, tumor microenvironmental factors such as hypoxia and inflammation often promote cancer progression so the effect of nutraceuticals on cancer cells exposed to such stress should be examined.

Plenty of experimental data support the tumor-suppressive effects of resveratrol, targeting malignant phenotypes of cancer cells. For example, expression of extracellular matrix (ECM)-degrading and remodeling enzymes like matrix metalloproteinase (MMP)-2 and MMP-9 are suppressed by resveratrol [10]. Resveratrol inhibits epithelial to mesenchymal transition (EMT) processes which are associated with chemoresistance and metastasis in multiple types of cancer [11,12,13,14]. These studies implicate that resveratrol can suppress metastasis by targeting multiple oncogenic pathways and regulate chemoresistance, invasion and migration of cancer cells in many adult cancer models including breast, lung, pancreatic, skin and prostate cancer models [13,15]. Additionally, suppression of cancer cell stemness by resveratrol has been reported, implying that this phytochemical can decrease the heterogeneity of a cancer cell population through the inhibition of the cancer stem cell population [16,17,18]. A growing body of evidence suggests that resveratrol exerts tumor-suppressive effects on neuroblastoma, which is a common extracranial solid tumor in children [19,20]. For example, resveratrol inhibited the growth of human neuroblastoma cancer cells (NGP and SK-N-AS cells) in mouse xenograft models [21]. Moreover, neuroblastoma cancer cells (NB-1691 cells) exhibited inactivation of AKT and increased cell death when resveratrol was co-treated with a glycolysis inhibitor, 2-deoxy-D-glucose (2-DG) [22]. Thus, resveratrol has tumor-suppressive potential on models of both adult and child cancers.

Recent studies have shown that resveratrol exerts tumor-suppressive effects, acting on both cancer cells and non-cancer cells of the tumor microenvironment. Non-cancer cells constituting the tumor microenvironment support cancer cells to survive under stressful conditions [23]. The current review highlights recent findings on resveratrol, which might serve as a tumor-suppressive therapeutic agent modulating the tumor microenvironment.

## 2. Resveratrol Modulating Signaling Pathways Activated by Stresses in Cancer Cells

**Figure 1:**
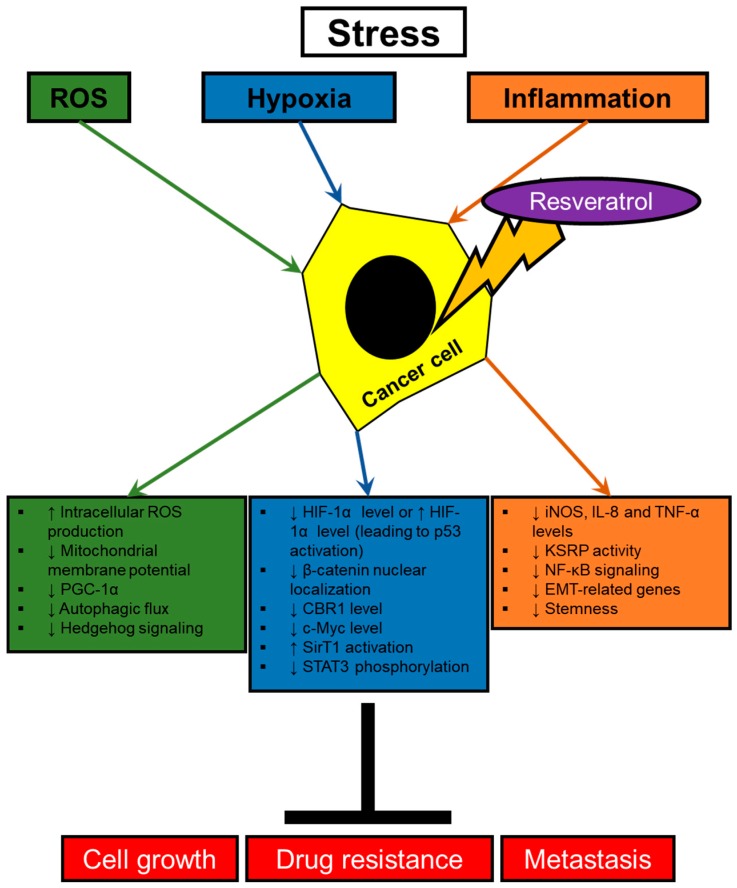
Tumor-suppressive effects of resveratrol targeting cancer cells in the tumor microenvironment. Various stresses in the tumor microenvironment affects cancer progression through signaling crosstalks. The effect of resveratrol on malignant phenotypes of cancer cells caused by tumor microenvironmental stress is summarized.

The tumor microenvironment is extremely dynamic and unstable. Cancer cells are exposed to various stress signals, which are associated with cancer progression. These stress conditions include hypoxia, oxidative stress and inflammation. Accumulating evidence shows that malignant properties are determined by the microenvironment that cancer cells are situated in. Hypoxia, oxidative stress and inflammation have been identified as positive regulators of metastatic potential, drug resistance and tumorigenic properties in cancer [24]. Recently, resveratrol has been suggested to suppress cancer progression stimulated by microenvironmental stress of the tumor (Figure 1).

### 2.1. Hypoxia

As metastasis and resistance to drug and radiotherapy are major culprits of poor prognosis in many types of cancer, understanding factors related to malignant phenotypes of cancer is crucial for devising anti-cancer strategies. Hypoxia is a hallmark of the tumor microenvironment correlated with poor prognosis in many cancer types [25,26,27]. The hypoxic area is created at the core region of a tumor distant from blood vessels, caused by the rapid proliferation of cancer cells and dysregulated angiogenic processes [28]. Under hypoxic conditions, cancer cells undergo epigenetic, transcriptomic and proteomic reprogramming, resulting in the acquisition of malignant phenotypes. Hypoxia-inducible factor-1α (HIF-1α) is a transcription factor that regulates expression of over 100 genes. HIF-1α gets stabilized under hypoxia, increasing aggressive phenotypes of cancer such as drug resistance and metastasis [29,30]. Additionally, recent findings suggest epigenetic modifications by G9a under hypoxia is involved in the malignant progression of cancer [31,32,33]. These cellular and molecular modifications enable cancer cells to survive under a hypoxic tumor microenvironment.

The cytotoxic effect of resveratrol on cancer cells under hypoxia has been reported. Castrated male BALB/cSlc-nu/nu mice bearing human prostate cancer cells (LNCaP cells) fed on a resveratrol diet (4 g/kg AIN-93G diet) showed significantly reduced tumor size, compared to the tumor of mice fed on a control diet. In tumors of mice fed on a resveratrol diet, HIF-1α expression was downregulated and nuclear localization of β-catenin was suppressed [34]. The hypoxic tumor microenvironment is associated with low pH conditions because hypoxia increases the rate of glycolysis, just followed by lactic acid production, making tumors more acidic than normal tissues. Interestingly, the cytotoxic effect of resveratrol is augmented in cancer cells under low pH conditions. Under low pH conditions, growth inhibition, internucleosomal DNA fragmentation and apoptosis were promoted after resveratrol treatment on human pancreatic cancer cell lines (Capan-2 and Panc-28 cells), but not on normal epithelial cells (HPDE cells). Taken together, resveratrol can target cancer cells exposed to low pH conditions [35]. This is a good example of taking advantage of the tumor microenvironment to eradicate cancer cells.

While a number of studies show that HIF-1α is negatively regulated by resveratrol, there is a report, showing resveratrol as a positive regulator of HIF-1α [36,37,38]. Treatment with resveratrol increased HIF-1α expression, triggering pseudo-hypoxic responses. This in turn, triggered p53 expression and apoptosis in murine prostate cancer TRAMP cells [38]. In general, cell death is induced when cancer cells are exposed to resveratrol. However, hypoxia signalings are affected in a context-dependent manner, depending on cells/tissues, so the mechanisms of action in response to resveratrol might be specific to cells/tissues.

Even synthetic small-molecule inhibitors designed to target the specific oncogenic signaling of cancer, show limited efficacy. So there could be limitations in using resveratrol as a naturally occurring compound and as a sole tumor-suppressive agent in clinical settings. Studies have been conducted to investigate the drug-sensitizing effect of resveratrol on hypoxic cancer cells when used in combination with other therapeutic agents [39,40]. Hypoxia induces doxorubicin resistance by increasing the expression of carbonyl reductase 1 (CBR1), an enzyme that converts doxorubicin into a less effective form, in cancer cells. Resveratrol downregulated HIF-1α expression occurs at the protein level, but not at the transcript level in human breast cancer cells (MCF-7 cells). Suppression of HIF-1α-induced CBR1 by resveratrol (10 µM) increased the sensitivity of hypoxic breast cancer cells to doxorubicin, making the hypoxic cancer cells sensitive to doxorubicin as shown in normoxic cancer cells [39]. Moreover, hypoxia-induced radioresistance is attenuated by resveratrol in human hepatoma cells (HepG2) through SirT1 activation and c-Myc downregulation, while resveratrol treatment did not alter the radiosensitivity of normoxic hepatoma cells [40].

Metastasis is associated with the poor prognosis of cancer and accumulative evidence proposes that hypoxia is an important driver of cancer metastasis. Studies have shown resveratrol can inhibit the metastatic progression of cancer cells enhanced by hypoxia. In human pancreatic cancer cells (BxPC-3 and Panc-1), resveratrol suppressed hypoxia-induced migration and invasion, downregulating HIF-1α protein expression [36]. Additionally, resveratrol decreased hypoxia-induced phosphorylation of STAT3 in human glioma cells (U251 and U87). A decreased p-STAT3 level by resveratrol led to an increase in the miR-34a level, suppressing migration and invasion of the cancer cells [41]. Human colon carcinoma cells (Lovo cells) treated with resveratrol showed a reduction in the HIF-1α protein level under hypoxia. Suppression of HIF-1α expression and stability by resveratrol decreased the metastatic potential of Lovo cells [42]. Metabolic alteration of cancer cells under hypoxia has been reported. Lysophosphatidic acid (LPA) is a phospholipid that is involved in the regulation of various cellular signaling. LPA is associated with malignant progression in many types of cancer [43,44,45,46]. Studies have suggested an increase in the LPA level in response to hypoxia [44,47]. As shown in the hypoxic tumor microenvironment, LPA is enriched in malignant ascites of patients with ovarian cancer as well. HIF-1α and vascular endothelial growth factor (VEGF) are upregulated by hypoxia-induced LPA signaling in human ovarian cancer cells (OVCAR-3 and CAOV-3). However, activation of HIF-1α and VEGF signaling pathways and subsequent migration of the ovarian cancer cells by LPA were attenuated by resveratrol [48]. Thus, resveratrol shows efficacy on cancer cells under hypoxia by modulating HIF-1α-related signaling pathways, resulting in induction of apoptosis, suppression of metastatic potential, such as migration and invasion and enhancement of drug sensitivity in many types of cancer.

### 2.2. Oxidative Stress

The excessive production of reactive oxygen species (ROS) and dysfunctional anti-oxidation machinery could result in increased oxidative stress in the tumor microenvironment. Cancer cells exhibit the increased intracellular level of ROS which is associated with constitutive activation of oncogenic signaling pathways [49]. Additionally, it has been well-documented that hypoxia and inflammation increase ROS production in many types of cancer [24]. The anti-oxidation capacity of resveratrol has been shown to be related to chemopreventive effect. Thus, scavenging ROS could be the key strategy for cancer prevention as elevated ROS induces DNA damage and malignant transformation of a cell [50].

In contrast, some studies have shown that cancer cells display increased intracellular ROS level when treated with resveratrol [51,52,53]. Human cancer cell lines (MCF-7, MDA-MB-231 and H1299 cells) showed an increase in the ROS level upon resveratrol treatment through the reduction in mitochondrial membrane potential and PGC-1α downregulation. Increased ROS by resveratrol promoted senescence of cancer cells through the DLC1-mediated FoxO3a/NF-κB signaling pathway [54]. Additionally, resveratrol synergetically induces cell death when treated with cisplatin in malignant human mesothelioma cells (MSTO-211H and H-2452 cells) by increasing the intracellular ROS level [55]. Resveratrol suppressed autophagic flux and increased the ROS level, causing cell death in human cholangiocarcinoma cells (QBC939 cells) [56]. Whereas, other studies demonstrated that resveratrol reduced oxidative stress in cancer. Treatment with resveratrol-loaded nanoparticles suppressed an increase in the ROS level by activation of the Nrf2-Keap1 signaling pathway in human lung cancer cells (A549 cells) [57]. Therefore, resveratrol could regulate the ROS level in a cell/organ type-specific manner. The resveratrol-delivery system could be developed and utilized for the effective treatment of cancer.

Cancer cells show a higher basal level of ROS than normal cells by intrinsic mechanisms of cancer cells as well as by external stimuli of the tumor microenvironment. As discussed above, hypoxia is one of the important environmental stressors stimulating ROS production. Hypoxia-mediated increases in ROS promotes migration and invasion of human pancreatic cancer cells (BxPC-3 and Panc-1 cells) through activation of the Hedgehog signaling pathway and this was reversed by resveratrol treatment [36].

Increased ROS production as a result of increased dependence on aerobic metabolism and decreased anti-oxidant gene expressions creates a ROS-rich tumor microenvironment, activating oncogenic signalings of cancer cells. The tumor-suppressive mechanisms by resveratrol, such as overproduction of ROS and subsequent activation of ROS-related signaling pathways could be utilized to develop anti-cancer strategies.

### 2.3. Inflammatory Signalings

Inflammation, a hallmark of the tumor microenvironment, is a major driver of cancer metastasis, associated with poor prognosis in many types of cancer. The inflammatory microenvironment of a tumor is caused by inflammatory cytokines including IL-1β, IL-6 and TNF-α [58]. These pro-inflammatory cytokines are upregulated and secreted not just by immune cells but also cancer cells and stromal cells adjacent to cancer cells in the tumor microenvironment, promoting cancer progression. TNF-α is a pleiotropic cytokine involved in pro-apoptotic and anti-apoptotic processes [59]. Despite its role in the induction of apoptosis, most cancer cells show resistance to TNF-α-induced apoptosis. Additionally, studies have shown that TNF-α increases the metastatic ability of cancer cells through activation of its downstream signaling pathways [60,61]. Also, other pro-inflammatory cytokines have been shown to induce malignant phenotypes of cancer such as chemoresistance and metastasis [62,63,64,65,66,67].

Dietary polyphenols have been studied on chronic inflammatory diseases. Resveratrol has shown its efficacy on chronic inflammation models [68,69]. The anti-inflammatory effect of resveratrol has been shown when a mixture of tetramethylpyrazine, curcumin and resveratrol suppresses inflammation in the collagen-induced arthritis rat model. A decrease in the serum level of pro-inflammatory cytokines including TNF-α, IL-1β and IL-6 was observed in the group that was given a combination of tetramethylpyrazine, curcumin and resveratrol [70]. Exposure of human colon epithelial cells (HT-29 cells) to a combination of cytokines (IL-1β TNF-α and IFN-γ) enhanced inflammatory signalings and ROS generation. Inflammation and ROS generation were reversed when the cells were pre-treated with resveratrol (25 µM) [71]. Resveratrol decreased mRNA levels of iNOS, IL-8 and TNF-α, mediated by selective binding to KH-type splicing regulatory protein (KSRP), which post-transcriptionally regulates pro-inflammatory genes [72]. Also, resveratrol elicits the tumor-suppressive effects, suppressing proliferation, migration and invasion in human colorectal cancer cells (HT-29 and HCT-116 cells). In tumor xenografts, injection with resveratrol markedly reduced IL-6 level [73]. These results indicate that resveratrol shows tumor-suppressive effects by blocking inflammatory responses of cancer cells.

Pro-inflammatory cytokines, which are rich in the tumor microenvironment can activate the NF-κB signaling pathway of cancer cells, increasing NF-κB nuclear translocation. Anti-inflammatory effects of resveratrol have been reported in different cells and tissues including brain and adipose tissues by modulating the inflammatory cytokine-mediated NF-κB signaling pathway [74,75]. In the study that used a co-culture model of macrophages and chondrocytes, resveratrol exerted anti-inflammatory effects by suppressing STAT3 and NF-κB signaling pathways. Using this model, they showed that IL-1β initiated inflammation of chondrocyte and inflammatory paracrine signaling of macrophages could be attenuated by resveratrol [76].

It is now well-recognized that activation of NF-κB signaling increases metastatic and survival of cancer cells [77]. Resveratrol inhibits TNF-α-induced migration and invasion of cancer cells through downregulation of NF-κB expression as well as its downstream molecules (uPA and uPAR) [78]. Also, TNF-β could enhance cancer cell survival and drug resistance. Resveratrol chemo-sensitized human colorectal cancer cells (HCT116 cells) to 5-FU under TNF-β inflammatory tumor microenvironment. Co-treatment of resveratrol with 5-FU suppressed NF-κB-mediated invasion, stemness, expressions of EMT-related genes in HCT116 cells exposed to the pro-inflammatory microenvironment [79]. Thus, oncogenic signaling pathways under inflammatory conditions could be mitigated by resveratrol.

## 3. Modulation of Angiogenesis by Resveratrol

Angiogenesis is a critical process for the proliferation and survival of cancer cells. For angiogenesis, stimulatory signals like growth factors and cytokines are necessary for the proliferation and migration of endothelial cells. Hypoxia and nutrient depletion caused by the rapid proliferation of cancer cells can limit tumor growth. To overcome this, cancer cells release pro-angiogenic cytokines, growth factors and exosomes required for survival and proliferation [80]. Anti-angiogenic effects of resveratrol could be exerted by targeting multiple oncogenic pathways in the tumor microenvironment.

### 3.1. Resveratrol Regulating Cytokine-Mediated Stimulation of Angiogenesis

Secreted proteins such as VEGF, basic fibroblast growth factor (bFGF) and IL-8 have been shown to stimulate migration and proliferation of endothelial cells to promote angiogenesis [81]. Resveratrol suppressed the secretion of IL-8/CXCL8 and VEGF in human peritoneal mesothelial cells (HPMCs). Incubation with conditioned medium obtained from resveratrol-treated HPMCs showed reduced proliferation and migration of human endothelial cells (HUVEC, HMVEC and HMEC-1 cells) [82]. Hepatic stellate cells, important cellular constituents of a liver, induce Gli-1 expression in human hepatocellular carcinoma cells (HepG2). Gli-1 expression triggers angiogenesis and ROS-induced metastasis. Treatment with resveratrol abrogated Gli-1 expression induced by hepatic stellate cells, suppressing angiogenesis and expressions of pro-angiogenic molecules (VEGF and CXCR4) in HepG2 cells [83]. Resveratrol enhanced anti-angiogenic and anti-proliferative effects of 5-FU, which is a chemotherapeutic agent widely used to treat cancer. Combined treatment with resveratrol and 5-FU exhibited a marked reduction in tumor growth and the formation of microvascular vessels in B16 murine melanoma tumor. Anti-angiogenic effects induced by co-treatment with resveratrol and 5-FU was correlated with increased p-AMPK expression. Moreover, vasodilator-stimulated phosphoprotein (VASP) and VEGF expressions were decreased in cancer cells in the B16 murine tumors co-treated with resveratrol and 5-FU [84]. Angiogenic responses promoted by activation of platelets was inhibited by resveratrol. Increased VEGF secretion of platelets by adenosine diphosphate (ADP) and lung cancer cells (A549 cells) was ablated by resveratrol [85]. These studies support that enrichment of pro-angiogenic factors could be blocked by resveratrol in the tumor microenvironment, thereby inhibiting blood vessel formation and tumor growth.

### 3.2. Effect of Resveratrol on Endothelial Cells

Endothelial cells are the main type of cells, forming a blood vessel wall. During angiogenesis, endothelial cells show a greater dependence on aerobic glycolysis [86]. Treatment with VEGF on human endothelial cells (HUVEC cells) increased expression of proteins involved in glycolysis such as glucose transporter 1 (GLUT1), hexokinase 2 (HK2), phosphofructokinase 1 (PFK1), pyruvate kinase M2 isoform (PKM2), inducing tube formation and migration. However, these effects were reversed by resveratrol through downregulation of the glycolytic genes, suppression of ERK1/2 and nuclear translocation of PKM2 [87]. A recent study proposes that resveratrol inhibits VEGF signaling through direct interaction with VEGF. Resveratrol suppressed proliferation, migration, invasion and the tube-forming capacity of endothelial cells, induced by VEGF. Blocking VEGF signaling by resveratrol decreased ROS generation and phosphorylations of VEGF receptor-2, JNK, eNOS, AKT and Erk, suppressing angiogenesis [88]. Both VEGF and bFGF are important stimulators of angiogenesis. Studies have shown that resveratrol suppresses angiogenesis through the inhibition of VEGF and bFGF [89,90]. Resveratrol inhibited the angiogenic response of murine endothelial cells (F-2 cells) promoted by VEGF. However, the bFGF-stimulated angiogenic response was not affected by resveratrol [91]. Intracellular accumulation of NO has been shown to play important roles in angiogenesis [92,93]. Nitric oxide (NO) generation induced by VEGF was abolished by resveratrol treatment in F-2 cells indicating resveratrol inhibits angiogenic responses by suppressing NO production. Whereas, bFGF did not affect NO production in F-2 cells. This suggests that resveratrol exerts an anti-angiogenic effect in a context-dependent manner when the endothelial cells are exposed to different angiogenic stimuli. [91]. Thus, resveratrol could be considered as an anti-angiogenic agent and has the potential to suppress cancer progression through the reduction of angiogenic responses and the secretion of pro-angiogenic factors.

## 4. Modulation of Non-Cancer Cells in the Tumor Microenvironment by Resveratrol

**Figure 2:**
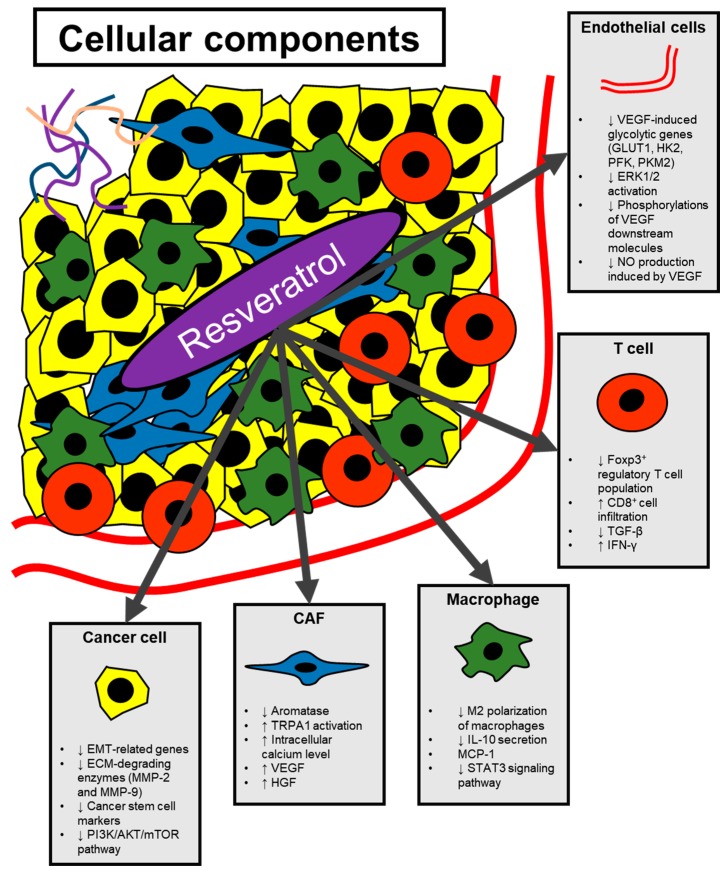
The effect of resveratrol on cells composing the tumor microenvironment. Cellular composition of tumor microenvironment affects cancer progression and oncogenic signaling. The effect of resveratrol on various cell types (cancer cells, CAFs, macrophages, T cells and endothelial cells) in the tumor microenvironment is displayed.

Tumors are composed of not just cancer cells but normal cells, which could affect malignant phenotypes of cancer. The major types of non-cancer cells that establish the tumor microenvironment are cancer-associated fibroblasts (CAFs), endothelial cells and immune cells like macrophages and T cells. Non-cancer cells in the tumor microenvironment educated by cancer cells undergo cellular reprogramming promoting metastasis and increase resistance to anti-cancer therapy in cancer cells [94,95,96]. Recent studies have revealed the impact of resveratrol on cellular components other than cancer cells in the tumor microenvironment (Figure 2).

### 4.1. Cancer-Associated Fibroblasts

Fibroblasts, commonly found in connective tissues, are the major cellular component of malignant tumors. They have specialized functions, such as the production of collagen fibers, mediation of the immune response to a tissue injury and the acceleration of cancer progression [97,98]. Among various sub-classes of fibroblasts, studies have shown that CAFs are closely related to tumor progression [99]. CAFs play a role in tumor metastasis, migration, tumor growth and drug resistance in the tumor microenvironment, mediated by secretory factors, extracellular matrix proteins and stimulatory molecules [100].

Resveratrol suppressed expression levels of cyclin D1, c-Myc, MMP-2, MMP-9 and Sox2, which were upregulated by CAFs in breast cancer cells. Also, resveratrol inhibited activation of AKT and STAT3 promoted by CAFs, thereby suppressing the expression of downstream molecules (CD44 and Sox2) associated with self-renewal in human breast cancer cells (T74D cells) [101]. Other researchers have investigated the effect of resveratrol on aromatase using a co-culture model of breast cancer cells with breast adipose fibroblasts (BAFs). Testosterone is converted to estradiol by BAFs, stimulating the proliferation of estrogen receptor-positive breast cancer cells. In the co-culture system, resveratrol suppressed the expression of pS2 and Ki67 upregulated by testosterone, inhibiting proliferation of human breast cancer cells (T47D cells) and BAFs [102]. However, when prostate cancer CAFs (PS30 cells) are exposed to resveratrol, they exhibit enhanced expression and secretion of oncogenic growth factors, HGF and VEGF. Activation of the transient receptor potential ankyrin 1 (TRPA1) channel by resveratrol which was found to be specific to PS30 cells harboring mutation at the N-terminal region of TRPA1 led to the accumulation of intracellular calcium in PS30 cells. Treatment with TRPA1 inhibitor (HC-030031) on PS30 abolished expression and secretion of VEGF and HGF increased by resveratrol. In the co-culture model of human prostate cancer cells (LNCaP cells) and PS30 cells, inhibition of TRPA1 by its inhibitor (HC-030031) sensitized LNCaP cells to resveratrol, shown by the increased apoptotic cell death rate [103]. Therefore, resveratrol could be used as a nutraceutical targeting CAF-enriched tumor microenvironment to increase the efficacy of other chemotherapeutic agents.

### 4.2. Macrophages

Macrophages are important cells of the immune system and are formed through the differentiation of monocytes. The polarization of macrophages into pro- and anti-inflammatory (M1 and M2) subtypes is affected by microenvironmental cues of tissue. In a tumor, the M1 to M2 ratio is an important factor that affects the prognosis in many types of cancer [104,105]. The M2 subtype of macrophages are often termed tumor-associated macrophages (TAMs). TAMs promote cancer progression and metastasis by releasing a number of oncogenic growth factors and cytokines. This is probably because of their essential role in the inflammatory response [106]. Therefore, studies on the relationship between cancer cells and TAMs are crucial for understanding mechanisms of cancer progression and metastasis in many types of cancer.

Accumulating evidence suggests that M2 macrophages activated by cancer cells could promote activation of STAT3, triggering secretion of several cytokines (IL-6 and IL-10) and this eventually causes increased tumor growth and metastasis [107,108]. Resveratrol facilitates anti-tumor effects by reducing the M2 polarization of human monocyte-derived macrophage (HMDMs). When the HMDMs were co-treated with resveratrol and tumor-conditioned medium (TCM), the secretion of IL-10, one of the M2-phenotype markers, was significantly lowered in comparison to the group only treated with TCM. Also, resveratrol significantly decreased STAT3 activation in cancer cells and tumor growth [109]. Furthermore, Kimura et al. carried out experiments to see the effect of resveratrol on the production of factors secreted from activated M2 macrophage and VEGF-C-induced capillary-like tube formation in human lymphatic endothelial cells (HLECs). Resveratrol showed anti-tumor and anti-metastatic effects by suppressing the expression of IL-10 and MCP-1 through downregulation of STAT3 signaling pathways. M2 polarization was inhibited by resveratrol. In addition, resveratrol treatment suppressed VEGF-C-induced HLEC motility, tumor growth and metastasis [110]. These studies suggest that resveratrol suppresses M2 polarization, inhibiting tumor progression and metastasis. Therefore, resveratrol could facilitate the tumor-suppressive effect through inactivation of TAMs.

### 4.3. T Cells

Resveratrol downregulates PI3K/AKT/mTOR pathway in cancer cells [111,112]. A study demonstrated that inhibition of the PI3K pathway activated CD8+ cytotoxic T cell activity, enhancing anti-cancer immunity [113]. This study suggested resveratrol might have the capacity to activate immune cells selectively inducing cell death in cancer cells. Regulatory T cells are abundant in solid tumors playing a pivotal role in regulating the homeostasis of the immune tumor microenvironment [114]. Increased numbers of regulatory T cells in the tumor microenvironment were negatively correlated with patient survival outcomes. Furthermore, regulatory T cells closely interact with other cells including CAFs, M2 macrophages, myeloid-derived suppressor cells and cancer cells in the tumor microenvironment [115,116,117].

Resveratrol has been shown to exert the anti-tumor effect through modulating the immune response in various cancer types. For example, Chen et al. [118] found the immunomodulatory effect of resveratrol on renal cell carcinoma (RCC). Resveratrol decreased regulatory T cells without the proportional change in myeloid-derived suppressor cells upon resveratrol treatment and increased the infiltration of activated CD8^+^ T cells in RCC tumors. Also, resveratrol promoted the expression of T-helper (Th)1 cytokine, IFN-γ, while attenuating the expressions of the Th2 cytokines, IL-6 and IL-10. Moreover, resveratrol was shown to have inhibitory effects on CD4^+^/CD25^+^ cell population among CD4^+^ cells through reduction of FoxP3, a specific regulatory T cell marker in tumor-bearing C57BL/6 mice. The mice injected with resveratrol exhibited a decrease in TGF-β production and an increase in IFN-γ production, which led to immune stimulation [119]. Additionally, resveratrol blocks progression and metastasis of breast cancer by inactivating tumor-evoked regulatory B cells (tBregs). Inactivation of tBregs by resveratrol suppressed the conversion of FoxP3^+^ regulatory T cells through the reduction of TGF-β production [120]. Taken together, these studies suggest that resveratrol modulates the immune tumor microenvironment, enhancing anti-cancer activity of immune cells.

## 5. Conclusions and Future Perspectives

Cellular components and stress conditions giving rise to complex and dynamic properties of the tumor microenvironment are closely associated with malignant phenotypes of cancer such as drug-resistance and metastasis. Until now, many studies have demonstrated the tumor-suppressive effect of resveratrol. Recently, it has become increasingly evident that resveratrol can also regulate the tumor microenvironment, suppressing cancer progression. Although the tumor microenvironment, which is composed of various cellular and non-cellular conditions, is extremely complicated, dissecting components of the tumor microenvironment affecting cancer cells into a single component might be necessary to better assess and understand the efficacy of resveratrol and other natural compounds. Once enough information on the effects of phytochemicals on the tumor microenvironment is gathered, recently developed drug-testing models could be utilized before proceeding to clinical trials. For example, a patient-derived xenograft (PDX) model, which can recapitulate complex constituents of the tumor microenvironment, could be incorporated to test the efficacy of resveratrol. Also, a patient-derived tumor organoid model, which is in vivo like the in vitro culture system could be used to test the effect of resveratrol. Through these approaches, personalized medicine could be achieved because tissues from patients with different clinical and molecular characteristics could be incorporated. Thus, considering heterogeneous environmental cues and cellular constituents of the tumor microenvironment, it is important to understand the effects of nutraceuticals on different types of cells in different microenvironmental conditions.

## Figures and Tables

**Table 1:** Information on resveratrol clinical trials in cancer.

Cancer Type	Sample Size and Phase	Dose	Status	Result	Year	Identifier	Ref.
Colon cancer	*n* = 11, phase 1	Resveratrol tablets; for 14 days, (80 mg/day or 20 mg/day) or grape powder (120 g/day or 80 g/day)	Completed	Expression of Wnt target genes was inhibited in normal colonic mucosa (*p* < 0.03), while Wnt target gene expression in colon cancer tissue was not altered by resveratrol/grape powder consumption. Consumption of grape powder (80 mg/day) showed the most notable decrease in Wnt target gene expression in normal colonic mucosa (*p* < 0.001).	From 2005 to 2009	NCT00256334	[7]
Colon and rectal cancer	*n* = 20, phase 1	Resveratrol; for 8 days prior to colorectomy	Completed	N/A	From 2006 to 2009	NCT00433576	N/A
Follicular lymphoma	*n* = 45, phase 2	Merlot grape juice 100 %; for 16 weeks, 660 mL or 495 mL every second day	Unknown	N/A	From 2007 to 2009	NCT00455416	N/A
Colorectal cancer and hepatic metastases of colorectal cancer	*n* = 9, phase 1	Oral administration of SRT501; 5.0 g/day for 14 days	Completed	Consumption of SRT501 (micronized resveratrol formulation) was well-tolerated. SRT501 showed better absorption and availability, compared to non-micronized resveratrol. A significant increase in caspase-3 expression by 39% was observed in malignant hepatic metastases.	From 2008 to 2009	NCT00920803	[8]
Multiple myeloma	*n* = 24, phase 2	Oral administration of SRT501; 5.0 g/day for 20 days	Terminated	Twenty-four multiple myeloma patients were treated with or without bortezomib. Since there was unexpected renal toxicity, the study was terminated early. Also SRT501 treatment showed minimal efficacy.	From 2009 to 2010	NCT00920556	[9]
Neuroendocrine tumor	*n* = 7, N/A	Oral administration of resveratrol; 5.0g/day for a total of three cycles	Completed	N/A	From 2011 to 2018	NCT01476592	N/A
Liver cancer	*n* = 0, Phase 1	Resveratrol; 1 g /day for 10 days prior to liver resection	Withdrawn	N/A	From 2015 to 2016	NCT02261844	N/A
Lymphangioleio-Myomatosis	*n* = 25, phase 2	Resveratrol;250 mg/day (first 8 weeks), 500 mg (next 8 weeks), 1000 mg/day for 8 weeks.	Recruiting	N/A	From 2018 to 2020 (estimated)	NCT03253913	N/A

Note: N/A denotes information not available.

## Abbreviations

2-DG	2-deoxy-D-glucose
ADP	Adenosine diphosphate
BAF	Breast adipose fibroblasts
bFGF	Basic fibroblast growth factor
CAF	Cancer-associated fibroblast
CBR1	Carbonyl reductase 1
ECM	Extracellular matrix
EMT	Epithelial-mesenchymal transition
GLUT1	Glucose transporter 1
HK2	Hexokinase 2
HLEC	Human lymphatic endothelial cell
HMDM	Human monocyte-derived macrophage
HPMC	Human peritoneal mesothelial cell
LPA	Lysophosphatidic acid
MMP	Matrix metalloproteinase
NO	Nitric oxide
PFK1	Phosphofructokinase 1
PKM2	Pyruvate kinase M2 isoform
RCC	Renal cell carcinoma
ROS	Reactive oxygen species
TAM	Tumor-associated macrophage
tBreg	Tumor-evoked regulatory B cell
TCM	Tumor conditioned medium
TRPA1	Transient receptor potential ankyrin 1
VASP	Vasodilator-stimulated phosphoprotein
VEGF	Vascular endothelial growth factor
